# Potential of *Azadirachta indica* as a Capping Agent for Antiviral Nanoparticles against SARS-CoV-2

**DOI:** 10.1155/2022/5714035

**Published:** 2022-09-15

**Authors:** Frank Eric Tatsing Foka, Nanabi Manamela, Steven Maluta Mufamadi, Hazel Tumelo Mufhandu

**Affiliations:** ^1^Department of Microbiology, Virology Lab, School of Biological Sciences, Faculty of Natural and Agricultural Sciences, North-West University, Mafikeng, Private Bag, X2046 Mmabatho, South Africa; ^2^Faculty of Health Sciences, Medical School, Nelson Mandela University, Missionvale Campus, P.O. Box 77000, Gqeberha 6031, South Africa

## Abstract

A rare type of pneumonia later on referred to as COVID-19 was reported in China in December 2019. Investigations revealed that this disease is caused by a coronavirus previously identified as SARS-CoV-2, and since then, it has become a global pandemic with new strains emerging rapidly as a result of genetic mutations. Various therapeutic options are being explored in order to eradicate this pandemic even though approved vaccine candidates are being currently rolled out globally. Most medicinal plant extracts have astonishing properties, and they can therefore be used in the biosynthesis of effective antiviral nanoparticles. In this systematic review, we aimed to highlight the specific attributes that make *Azadirachta indica* (neem plant) a suitable candidate for the biosynthesis of anti-SARS-CoV-2 nanoparticles. A systematic investigation was therefore carried out in PubMed, Scopus, Web of Science, and AJOL databases with the keywords “Nanoparticles,” “Biosynthesis,” “Antivirals,” “SARS-CoV-2,” and “*Azadirachta indica*.” 1216 articles were retrieved by the 21^st^ of February 2022, but we screened studies that reported data on biomedical and antimicrobial assessment of *Azadirachta indica* extracts. We also screened studies that were reporting nanoparticles possessing antiviral properties against SARS-C0V-2, narrowing our results to 98 reports. Herein, the SARS-CoV-2 viral structure is briefly discussed with nanoparticles of biomedical importance in the design of SARS-CoV-2 antivirals. Most importantly, we focused on the biomedical and antiviral properties of *Azadirachta indica* extracts that could be of importance in the design of potential anti-SARS-CoV-2 nanoformulations.

## 1. Introduction

The city of Wuhan in Hubei Province, China, was the first site of a pneumonia outbreak linked with the novel coronavirus, SARS-CoV-2. The virus erupted into a global pandemic causing a disease named coronavirus disease 2019 (COVID-19) that affects people differently with some being severely affected while others have mild or no symptoms [1]. It has been established that underlying or chronic diseases as well as advanced age are predisposing factors for the development of severe symptoms or complications [2]. Symptoms range from mild flu-like symptoms such as headache, fever or chills, congestion or runny nose, fatigue, muscle or body ache, to severe life-threatening pneumonia with difficulty in breathing, chest pains, loss of taste, loss of smell, nausea and vomiting, and diarrhea with sore throat and dry cough [1]. In order to curb this pandemic, repurposing of certain existing medicines was attempted as a treatment option. Undeniably, bromhexine, fluoxetine, molnupiravir, and paxlovid have shown promising results as add-on therapies and the Emergency Use Authorization (EUA) of remdesivir, dexamethasone, and monoclonal antibodies has also shown significant results [3–8]. Thus far, these treatment options prove to be less efficient [8]. The World Health Organization (WHO) has approved several vaccines against COVID-19; however, they seem to show lower efficacy against new emerging variants [9]. These variants of concern can evade the immune system, show increased transmissibility, and cause severe disease [10], causing breakthrough infections and reinfections [11]. Thus, there is a dire need to find effective antivirals to control the spread of COVID-19 [12].

The use of nanomaterials to design and develop antiviral substances has been previously explored as they can be directly delivered to the site of infection (to the lungs in the case of COVID-19) to effectively attack viruses [12–15]. Therefore, nanotechnology has emerged as an attractive therapeutic strategy for many infectious diseases. Besides the fact that plants are a primary food source, they are also important medicinal alternatives in combatting diseases. Plants produce various secondary metabolites that are involved in their self-defense mechanisms against stress-induced environmental factors and pathogens [16]. Such metabolites extracted from medicinal plants have been used in traditional herbal medicine for centuries. Hence, there is a plethora of identified and well-characterized medicinal plants whose extracts or raw parts are used to treat diseases as well as infections caused by respiratory viruses including SARS-CoV-2 [17–20]. *Azadirachta indica*, commonly known as neem, is one of such medicinal plants which displays antiviral and immunomodulatory activities against Herpes Simplex Virus type 1 and SARS-CoV-2 [21].

Biosynthesis of nanoparticles (NPs) is an eco-friendly method used to produce NPs; it is easy and cost-effective with minimal impacts on the environment. Moreover, the various polyphenols and proteins that are contained in the plants act as reducing agents which makes the biosynthesis process less toxic when compared to metallic compounds which may be hazardous [20]. However, metallic NPs capped with medicinal plant extracts could be promising antiviral agents with high efficacy. Therefore, this review's main aim is to point out, through a rigorous literature analysis and a clearly defined methodology, the latest findings from studies assessing nanoparticle-based assays against coronaviruses until 2022, but more specifically, this review highlights the properties of *Azadirachta indica* which make it a suitable candidate for the biosynthesis of antiviral NPs against SARS-CoV-2.

## 2. Search Methodology

This systematic review retrieved and analyzed data on nanoparticles and *Azadirachta indica* extract biomedical properties with emphasis on those that have antiviral activities against viruses and coronaviruses from literature databases. No time restriction for the publications was set, and PRISMA guidelines were followed to retrieve and analyze the data as described previously [22].

### 2.1. Research Enquiry and Search Question Formulation

“Problem, intervention, comparison, outcome, and study type (PICOS)” procedure was used to draft the research question. We took into consideration molecular docking studies, clinical and preclinical investigations, and hypothetical and theoretical studies. The research questions were therefore formulated as follows: what are the potential nanoparticles that are virucidal on coronaviruses? What are the antiviral properties of *Azadirachta indica*? Which *Azadirachta indica*-capped nanoparticles demonstrated virucidal activities on viruses and coronaviruses so far?

### 2.2. Databases of Data Retrieval and Assessment

We browsed through the following databases: PubMed, Scopus, Web of Science, AJOL, and ScienceDirect. The reference lists from relevant manuscripts were also assessed for reports that might be of interest. The search strings were determined by initially defined keywords such as coronaviruses, nanoparticles biosynthesis, and *Azadirachta indica*.

### 2.3. Criteria for the Selection of the Reports

**Figure 1:**
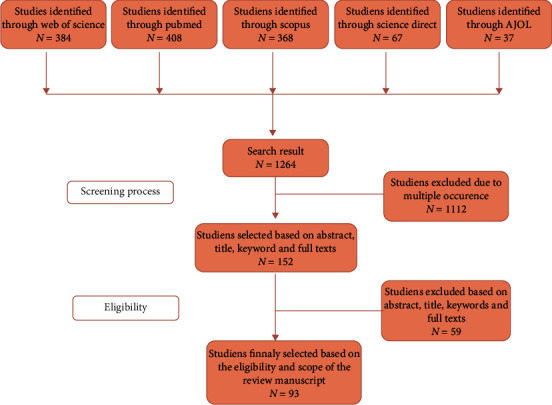
Flow diagram of the studies used in this systematic review.

Only studies written in English were considered for this report. No date or time restriction was set, the search was initiated on August 2021, and an update was made on the 23^rd^ of December 2021 and the 21^st^ of February 2022 for a maximum retrieval of relevant manuscripts. Thesis, abstracts, meetings, and studies that were irrelevant to the topic of research were excluded in this systematic review (Figure 1).

### 2.4. Data Assessment and Probable Sources of Bias

The authors went through the titles and abstracts of the selected reports to be certain that they were within the scope of this systematic review. Full-text manuscripts were downloaded; all sections (abstract, introduction, methodology, results, conclusion, references, tables, and figures) were scrutinized independently for data gathering. The only probable sources of bias might be our criteria of selection and possibly data omission.

## 3. Results

As illustrated in our PRISMA flow diagram, our systematic investigation yielded 408 publications in PubMed, 384 in Web of Science, 368 through Scopus, 67 through ScienceDirect, and 37 through AJOL, making a total of 1264 research papers. Out of these, 1112 were either reoccurring information or studies that were irrelevant with regard to our topic and were therefore excluded. Ninety-eight studies were finally selected after a thorough assessment of the titles, abstracts, and full texts as they were in line with our criteria of eligibility for this manuscript. Of these 93 manuscripts, structure and genetic features of SARS-CoV-2 were discussed extensively in 22 studies; meanwhile, nanoparticles of biomedical importance in the design of SARS-CoV-2 antivirals were discussed in 21 studies. Furthermore, 50 manuscripts discussed about the biomedical properties of *Azadirachta indica* (neem) extracts with emphasis on their antiviral features.

## 4. Exploratory Analysis of the Data Recovered in the Literature

### 4.1. Features and Genetic Attributes of SARS-CoV-2

Coronaviruses are positive, single-stranded RNA viruses [23]. They belong to the Orthocoronavirinae subfamily under the Coronaviridae family, which has four genera, namely, Alphacoronavirus, Betacoronavirus, Deltacoronavirus, and Gammacoronavirus [24]. Guo et al. indicated that sources of alpha and beta coronaviruses are rodents and bats, while those of delta and gamma coronaviruses are birds [25]. Coronaviruses are zoonotic and lead to conditions such as neurological, respiratory, hepatic, and enteric diseases in many animals and cause acute infections such as the common cold and potentially fatal severe respiratory tract infections in humans [26, 27]. Thus far, there are seven known human coronaviruses, HCoV-OC43, HCoV HKU1 which are Betacoronaviruses of the A lineage and HCoV-229E and HCoV-NL63 which are Alphacoronaviruses with low virulence and result in mild respiratory infections [25, 28]. The Betacoronaviruses of the B lineage and the Middle East respiratory syndrome coronavirus, a Betacoronavirus of the C lineage, are responsible for severe acute respiratory syndromes (SARS-CoV, MERS-CoV, and SARS-CoV-2, respectively) [27–29]. SARS-CoV-2 was announced by WHO as a health emergency on the 30th of January 2020 and as a pandemic on the 11th of March 2020; it has claimed 6,142,735 lives globally and infected 486,761,597 people as of the 2^nd^ of April 2022 (WHO, 2022). The virus has undergone genetic evolution and mutated, giving rise to several variants of concern which have spread globally (Table 1).

SARS-CoV-2 can be transmitted directly or indirectly from animals and infected individuals to humans [21, 30]. Direct transmission occurs when people are in close contact with an infected individual and are exposed to respiratory fluids by inhaling airborne aerosols from an infected person. Indirect transmission occurs by touching infected surfaces and then transferring the fluids to the mucous membrane of the eyes, mouth, or nose [1]. Signs of infection by SARS-CoV-2 differ between individuals and may range from mild symptoms to complications like severe respiratory failure [30]. Besides the symptoms mentioned above, some patients may experience a cytokine storm which may lead to poor blood oxygenation and acute respiratory distress syndrome (ARDS), which in 20-50% of cases leads to death [31].

SARS-CoV-2 genome is 29.9 kb long and has open reading frames (ORFs) which synthesizes structural and nonstructural proteins [32–34]. Two-thirds of the genome, the ORF1a and ORF1b, encode the replicase genes which are translated and processed into 16 nonstructural proteins. Four structural proteins including envelope (E), membrane (M), nucleocapsid (N), and spike (S) with accessory proteins make up the other third of the genome [35]. The spike protein connects to the angiotensin-converting enzyme 2 (ACE2) receptor on the host cell surface, and it is split by the transmembrane protease serine 2 into two subunits (S1 and S2) and this cleavage enhances viral entry [35, 36].

Reverse transcription polymerase chain reaction (RTPCR) is used, as indicated by the WHO, for the detection of active SARS-CoV-2 infection from nasopharyngeal or oropharyngeal samples. Since the pandemic started, several vaccines have been approved to mitigate the spread of COVID-19 [37]. The vaccines that have been approved so far are safe and effective even in individuals with underlying medical conditions who are at higher risk of severe sickness. However, the pandemic is still a major public health concern even in the presence of different vaccine options [38]. Variants of concern are posing serious challenges as they cause severe disease, show increased transmissibility, and can evade the immune system. Thus, they result in people being reinfected and leading to breakthrough infections [10]. On the other hand, there is a dilemma of vaccine hesitancy as people are concerned about their effectiveness and impact on their overall health [39]. Thus far, COVID-19 is managed by prescribing medication to address clinical symptoms and by using mechanical ventilation to support the respiratory systems of those who are severely ill [27]. The development of safe, biocompatible treatment options that will inhibit the virus and combat its spread will be a tremendous advantage for humanity.

### 4.2. Nanoparticles of Biomedical Importance in the Design of SARS-CoV-2 Antivirals

Since the discovery of their potential for biomedical applications, NPs have attracted the attention of the research community, which is restlessly investigating their biocidal capabilities and potentially effective formulations. Nanomaterials may be used to contain the spread of viruses as some have antiviral activities and are able to be delivered and released slowly in the body [12, 14]. According to Jeevanandam et al., nanomaterials are materials with a varying size of 1-1000 nm in at least one dimension [40]. They have unique properties including appropriate size, ideal shape, surface charge that can be tuned, superparamagnetism, increased surface plasmon resonance, luminescence, bioavailability, biocompatibility, immunocompatibility, tolerability, biodegradability, and photon upconversion [41]. Since metal ions are vital for living beings, metal-based nanomaterials are used for various applications in the biomedical field including the development of antivirals [7].

**Figure 2:**
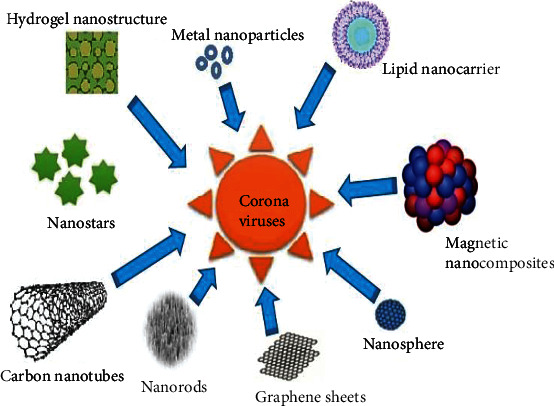
Shapes and structures of nanoparticles in formulations against coronaviruses.

NPs are considered as innovative tools that can enhance the delivery of various therapeutic formulations such as vaccines and drugs, because of characteristics such as size, charge, solubility, ease of synthesis, biocompatibility, biodistribution, bioavailability, biodegradability, slow-release mechanism, and enhanced retention inside target tissues [12, 42]. Several types of nanostructures have been synthesized as antiviral substances against SARS-CoV-2 (Figure 2).

These include nanorods, nanotubes, nanospheres, nanocrystals, nanowires, nanosheets, nanostructured lipid carriers (NLCs), and nanogels. Almanza-Reyes and colleagues demonstrated that silver nanoparticles (Ag NPs) were able to inhibit SARS-CoV-2 infection both *in vitro* and *in vivo* among randomized healthcare personnel [43]. Another molecular docking study by Attia et al. highlighted the inhibitory effects of zinc oxide nanoparticles (ZnO NPs) capped with hesperidin on SARS-CoV-2 [44]. Antiviral metal nanoparticles bind to the surface of viruses, preventing them from attaching to host cell receptors and can interfere with the early stages of viral replication and suppress free virions [45]. Moreover, nanomaterials such as Ag NPs have immunomodulatory characteristics which may considerably help to prevent the development of a cytokine storm [13, 20]. Additionally, Skalny et al. indicated that the current data on zinc prove that its ions have anti-inflammatory activity in pneumonia, thereby inhibiting tissue damage and its systemic activity inhibits respiratory syncytial virus replication [46]. This encouraged physicians to prescribe zinc-containing compounds in the COVID-19 protection protocol. Fouad reported that the zinc oxide nanoparticles (ZnO NPs) are nontoxic, less costly, biocompatible, and antioxidant and have anti-inflammatory activity [47]. Nanomaterials also have inhibitory effects on many other viral pathogens as highlighted by several studies (Table 2). For example, several studies proposed that silver nanoparticles are effective against Herpes Simplex Virus type 1 [48], HIV-1, Respiratory Syncytial Virus [49], and Influenza A virus [19]. Fouad also demonstrated in his study that ZnO NPs can inhibit the H1N1 influenza virus [47]. They interfere with the replication, translation, and release of the virus [50]. According to Attia et al., silver sulfide nanoclusters display positive antiviral activity against the viral replication of Porcine Endemic Diarrhea Virus (PEDV), a positive-strand RNA virus [44]. This is also supported by Du et al. who inferred in their study that glutathione-encapsulated silver sulfide nanoclusters possess antiviral characteristics that prevent synthesis and budding of PEDV [49]. In the same order of ideas, copper nanoparticles (Cu NPs) coupled with polymers were proven to have biocidal effects on coronaviruses through an oxidative stress which is enhanced by a sustainable release of bioactive copper ions as the nanocomposite is absorbed on the microorganism's surface [51].

FDA authorized the repurposing of ferric oxide (Fe_2_O_3_) and ferrosoferric oxide (Fe_3_O_4_) magnetic nanoparticles in the control of COVID-19 as a result of their ability to repel water and hydrogen binding with the SARS-CoV-2 S receptor, leading to inability of the virion to attach to the host cell receptor. Similarly, it was proven that functionalized gold nanoparticle (Au NP-) and silica nanoparticle- (SiNP-) encapsulated PolyP prevent binding to the host cell [52, 53]. Moreover, another research team used nanoformazans as antivirals to reduce COVID-19 infection through the inhibition of viral replication [54]. Theoretical approaches, preclinical studies, hypotheses, and extensive research outputs on recent NP developments for various methods of detection and control of SARS-CoV-2 are extensively elaborated in a previous study by Carvalho and Conte-Junior [12].

### 4.3. Biomedical Properties of *Azadirachta indica* (Neem) Extracts

**Figure 3:**
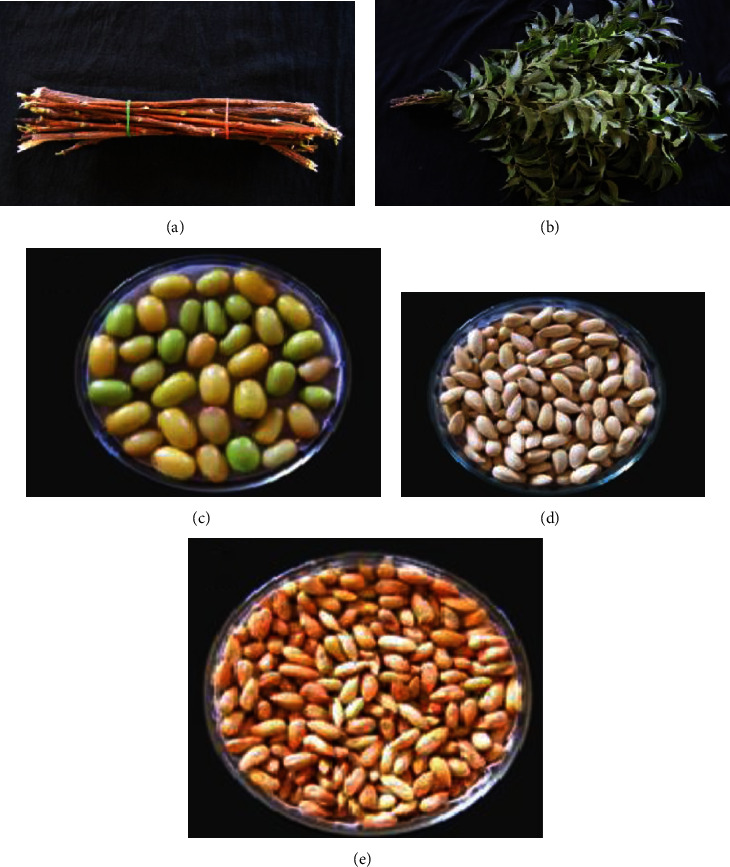
Neem plant parts and products: (a) twigs, (b) leaves, (c) fruits, (d) seeds (with endocarp), and (e) seeds (without endocarp).

*Azadirachta indica* (neem) belongs to the Meliaceae family which is commonly found in tropical Africa, Pakistan, Nepal, Bangladesh, and India. It is a tree that grows fast, reaching 20 to 23 m in height with a trunk diameter of 4 to 5 feet. It produces green drupe fruits that become golden yellow when ripe (Figure 3) [55]. Neem parts such as the leaves, fruits, seeds, and bark of the tree contain various active ingredients that have therapeutic properties against a wide variety of disease conditions.

Azadirachtin is the one of the most important active compounds found in this plant. It is a tetranortriterpenoid limonoid which is present mostly in the seeds. Among other active ingredients of biomedical importance, one can cite quercetin, salannin, gedunin, sodium nimbinate, nimbidol, nimbolinin, nimbinin, nimbidin, and nimbin (Table 3). Some of the substances of biomedical importance extracted from the leaves include 6-desacetylnimbinene, nimbidol, 17-hydroxyazadiradione, nimbin, 7-desacetyl-7-benzoylgedunin, nimbandiol, ascorbic acid, nimbolide, n-hexacosanol, and 7-desacetyl-7-benzoylazadiradione (Table 3).

**Figure 4:**
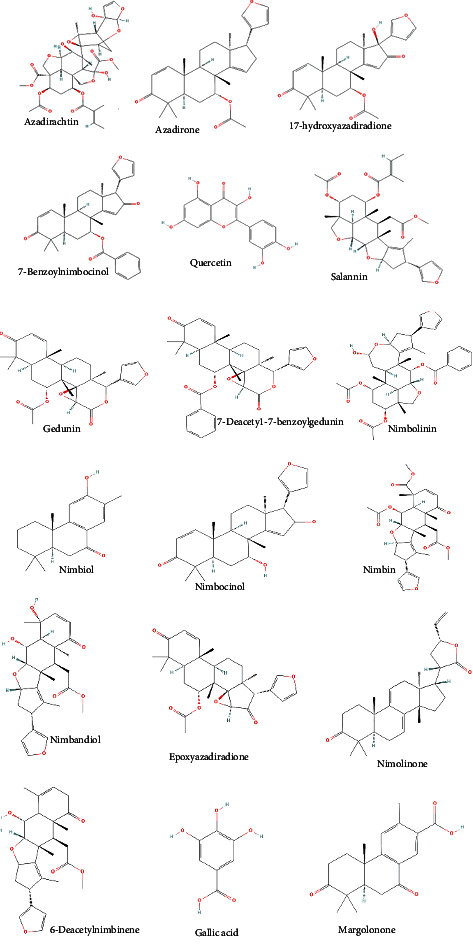
Chemical structures of *Azadirachta indica* bioactive compounds.

*Azadirachta indica* extracts have inhibitory effects on microbial growth through the initiation of cell membrane lysis. Plant parts and products such as the seeds oil, the fruits, the bark, and the leaves are vital in preventing disease because of their high content in antioxidants. Several studies have demonstrated the free radical scavenging properties of azadirachtin and nimbolide due to their chemical structure (Figure 4) [56, 57, 75]. Additionally, neem seeds oil is effective against cancer cell proliferation as its constituents activate tumor suppressor genes and inactivate genes that are involved in cancer proliferation [76]. It also enhances apoptosis, elimination of NF-*κ*B signaling, and the cyclooxygenase pathway [55, 72, 75]. Furthermore, *Azadirachta indica* leaves, seeds, and bark extracts have anti-inflammatory properties [56, 57, 73], antipyretic and antidiabetic properties [77], hepatoprotective effects and wound healing properties, neuroprotective effects, immunomodulatory and growth promoting effects, and antinephrotic properties [55, 78–80].

Most importantly, neem extracts display antimicrobial activities. For instance, in a previous study, leaf extracts demonstrated antibacterial properties far greater than those of sodium hypochlorite [81, 82]. Moreover, in an experiment designed to investigate the effects of neem leaf extract on seed-borne fungi *Aspergillus* and *Rhizopus*, the results demonstrated that the fungi were significantly controlled with alcoholic and water extracts of neem [83]. In the same way, neem bark extracts are antimalarial with a proven effect on both sexual and asexual forms of *Plasmodium falciparum*; other studies have also demonstrated that limonoids and specifically azadirachtin from neem seed extracts are effective on malaria vectors [58, 84–86].

The burden of the COVID-19 pandemic has motivated the scientific community to investigate on safer and efficient therapeutic options in order to combat the disease. Consequently, because of its eco-friendly aspect, green synthesis of nanoparticles from natural plants has gained attention in pharmaceutical applications. As far as SARS-CoV-2 is concerned, most studies highlighting the antiviral effect of neem plant extracts on this pathogen are molecular docking studies. These studies are yet to be carried out in vitro and in vivo, and producing biosynthesized NPs capped with *Azadirachta indica* extracts would be a good option to explore for the design of nanoformulations against SARS-CoV-2. Scientific reports highlighted the inhibitory effects of neem extracts on the SARS-CoV-2 as well as their antiretroviral properties [87].

Neem bark extracts demonstrated antiviral and immunomodulatory activities against Herpes Simplex Virus type 1 and SARS-CoV-2 [60, 69, 73, 88, 89]. Furthermore, a study assessing the inhibitory effect of 160 phytocompounds demonstrated that azadirachtin was highly effective against SARS-CoV-2 through the inhibition of Mpro, PLpro, and RdRp proteins, respectively [90]. Additionally, in vivo intranasal and oral administration of nimbin and its isomers extracted from neem bark demonstrated inhibitory responses towards m-CoV-RSA59 and SARS-CoV-2 by inhibiting viral expression and spread [91]. Similarly, another study demonstrated the inhibitory effect of azadirachtin, quercetin, and margocin on the cell entry by SARS-CoV-2 through the blocking of RBD-ACE2 spike receptors [92]. In the same way, scientific investigations also demonstrated antiviral effects of neem extracts on smallpox virus, chickenpox virus, polio virus, dengue virus, and HIV [86, 88, 89]. Another molecular docking study by Baildya et al. proposed the potency of neem seed extract against SARS-CoV-2 [69]. In summary, these properties make the neem extracts suitable candidates and capping agent for the biosynthesis of NPs that can be used as antivirals or antimicrobial formulations against infectious pathogens such as SARS-CoV-2. This area of research is yet to be fully explored. The properties of this medicinal plant are remarkable and provide a better perspective for the future in terms of infectious pathogen control.

## 5. Conclusion

It is quite evident that the SARS-CoV-2 pandemic is one of the biggest threats to human health the world has ever witnessed. As a result, developing new, efficient, and cost-effective antiviral therapeutics is a research priority. The green synthesis of nanoparticles should be strongly encouraged as the search for therapeutics against SARS-CoV-2 continues. Although issues regarding the level of toxicity and the impact of medicinal plant extracts on vital organs are not to be undermined, plant extracts remain suitable, safer, and effective multicomponent mixtures in the development of therapeutic formulations against infectious pathogens. The neem plant extracts have been shown to possess multiple therapeutic effects, and their potential in the formulation of virucidal nanoformulations against SARS-CoV-2 and other viruses is yet to be fully explored. Indeed, the physicochemical and biomedical properties of *Azadirachta indica* (neem) extracts make this plant an ideal candidate for the biosynthesis of effective biological NPs against SARS-CoV-2 and many other pathogens.

## Figures and Tables

**Table 1:** Molecular and clinical features of SARS-CoV-2 variants of concern.

		Variants of concern				
		Alpha	Beta	Gamma	Delta	Omicron
Pango lineages		B.1.1.7	B.1.351 B.1.351.2 B.1.351.3	P.1 P.1.1 P.1.2	B.1.617.2 AY.1 AY.2 AY.3	B.1.1.529 BA.1 BA.2 BA.4 BA.5

Epidemiology	Date and place of initial detection	September 2020, UK	May 2020, South Africa	November 2020, Brazil	October 2020, India	November 2021, Botswana
	Date of designation	December 2020	December 2020	January 2021	VOI: April 2021 VOC: May 2021	VOC: November 2021
	Global spread	48%	7%	7%	14%	43%
	Geographic localization	Worldwide	South Africa	South America	Asia	Worldwide

Predominant mutations		N501Y D614G, P681H	K417N, E484K, N501Y D614G	K417T, E484K, N501Y D614G	L452R, E484Q, T478K D614G, P681H	N501, H69-, Y453F, L18, K417, P681, E484, Q677, S477, Y144-, H655, S3675

Phenotypic impacts	Transmissibility	(i) High transmissibility (ii) High rate of secondary attack	(i) High transmissibility	(i) High transmissibility	(i) High transmissibility (ii) High rate of secondary attack (iii) High transmissibility between vaccinated and unvaccinated individuals	(i) High transmissibility
	Disease severity	(i) Increased risk of hospitalization (ii) Increased risk of severity (iii) Increased risk of mortality	(i) Increased risk of in-hospital mortality	(i) Possible increased risk of hospitalization	(i) Increased risk of hospitalization	(i) Possible risk of hospitalization
	Risk of reinfection	(i) Neutralizing activity retained (ii) Risk of reinfection remains similar	(i) Reduction in neutralizing activity reported (ii) T cell response remains effective	(i) Moderate reduction in neutralizing activity reported	(i) Reduction in neutralizing activity reported	(i) Reduction in neutralizing activity reported
	Diagnosis	(i) Limited impact—S gene target failure (ii) No impact on overall result from multiple target RT-PCR (iii) No impact on AgRDTs observed	(i) No impact on RT-PCR or AgRDTs observed	(i) None reported to date	(i) None reported to date	(i) S-assay within TaqPath tests gives negative results (ii) No impact on result from multiple target RT-PCR

**Table 2:** Anticipated approaches in predicting ligand-receptor binding and drug structures for COVID-19 management.

NPs	Dimension	Method	Ligand-receptor binding information	Potential application	References
Iron oxide nanomaterials	NR^a^	Nanostructure of Fe2O3^b^ and Fe3O4^c^	Interactions with S1-RBD^d^ of SARS-CoV-2^e^	Repurposing medication	[12]
PolyP^f^/silica nanoparticle	210 ± 40 nm	Optimized polyP^f^ encapsulated by SiNPs^g^	Inhibition of binding of ACE2^h^ to S-protein SARS-CoV-2, at a physiological solution	Immunologic agents	[52]
Gold nanoparticles	NR	Peptide-functionalized gold nanoparticles	More stable complex with RBD of SARS-CoV-2 than ACE2	Antiviral agents	[53]
Nano-sized formazans	23.75 ± 7.16 nm	Formazan analogs by dithizone and *α*-haloketone reaction	Inhibition of SARS-CoV-2 chymotrypsin-like protease, at a physiological solution	Antiviral agents	[54]
L-PLGA NPsi	NR	Optimized remdesivir-loaded L-PLGA NPs^i^	Interactions lisinopril-ACE1^g^ and remdesivir intracellular targeting protein RdRp^j^	Antiviral therapy	[12]
Silver nanoparticles	NR^a^	Artemisinin, artemether, and artesunate delivery by silver nanoparticles	Interactions between negative charges of oxygen atoms of drugs with Ag surface	Antiviral drugs	[27]

^a^Not reported; ^b^iron (II) oxide or magnetite; ^c^iron (II,III) oxide or hematite; ^d^chimeric spike-receptor-binding domain; ^e^novel coronavirus; ^f^polyP; ^g^silica nanoparticle; ^h^angiotensin-converting enzyme inhibitor 1 or 2; ^I^lisinopril covalently grafted onto poly(lactic-*co*-glycolic acid) nanoparticles; ^j^RNA-dependent RNA polymerase.

**Table 3:** Biomedical properties of some chemical substances contained in *Azadirachta indica* extracts.

Compound name	Plant source	Biomedical properties	References
Azadirachtin	Seeds, bark	Antitumor, antiviral, antimalarial	[56, 57]
Azadirone	Seeds, bark	Antimalarial, insecticidal	[58, 59]
17-Hydroxyazadiradione	Leaves	Antiviral, antimalarial, antifungal	[60, 61]
7-Desacetyl-7-benzoylazadiradione	Leaves	Antidiabetic	[62]
Quercetin	Seeds	Antifungal, antibacterial, antiviral	[63]
Salannin	Seeds	Insecticidal	[64]
Gedunin	Seeds	Antimalarial, antifungal, antiviral, and antiparasitic	[65–67]
7-Deacetyl-7-benzoylgedunin	Leaves	Antiviral	[65]
Nimbidin	Seeds	Anti-inflammatory	[57]
Nimbolinin	Seeds	Antibacterial, anti-inflammatory	[56, 68]
Nimbiol	Seeds, leaves	Antiviral	[69–71]
Nimbocinol	Seeds, leaves	Antiviral	[69–71]
Nimbidol	Seeds, leaves	Anti-inflammatory	[57, 72]
Nimbandiol	Leaves	Antiviral	[60]
Nimbin	Seeds, leaves	Antiviral, anti-inflammatory, insecticidal	[64, 73, 74]
Nimbinin	Seeds	Antiviral	[66]
Nimbolide	Leaves	Antimalarial, antibacterial	[56, 57, 75]
Nimolinone	Seeds, leaves	Antitumor	[76]
6-Deacetylnimbinene	Leaves	Antitumor	[76]
N-Hexacosanol	Leaves	Antidiabetic	[77]
Ascorbic acid	Leaves	Immunomodulator	[78, 79]
Gallic acid	Bark	Antibacterial, antiviral, antioxidant, immunomodulator	[63, 79–81]
Catechin	Bark	Antibacterial, antiviral	[79, 81]
Margolonone	Bark	Antiviral, antibacterial	[60, 81]
